# Ultrasound-guided percutaneous radiofrequency ablation combined with anti-PD-1 for the treatment of prostate cancer: an experimental study

**DOI:** 10.3389/fonc.2025.1527763

**Published:** 2025-03-24

**Authors:** Si Chen, Ruiqing Liu, Shaobo Duan, Beibei Zhang, Yuzhou Wang, Xiaoxiao Li, Yingying Zhao, Zesheng Li, Qi Zhou, Rui Zhang, Linlin Zhang, Xiaoxia Xu, Ru Jang, Juan Zhang, Yaqiong Li, Xiguo Cai, Lianzhong Zhang

**Affiliations:** ^1^ Zhengzhou University People’s Hospital, Henan Provincial People’s Hospital, Zhengzhou University, Zhengzhou, Henan, China; ^2^ Department of Interventional Therapy, Henan Provincial People’s Hospital, Zhengzhou, Henan, China; ^3^ Department of Ultrasound, Henan Provincial People’s Hospital, People’s Hospital of Zhengzhou University, Henan University People’s Hospital, Zhengzhou, Henan, China; ^4^ Henan Provincial International Joint Laboratory of Ultrasonic Nanotechnology and Artificial Intelligence in Precision Theragnostic Systems, Henan Provincial People’s Hospital, Zhengzhou, Henan, China; ^5^ Henan University People’s Hospital, Henan Provincial People’s Hospital, Henan University, Zhengzhou, Henan, China; ^6^ Henan Provincial Clinical Research Center for Rehabilitation Medicine, Henan Provincial People’s Hospital, Zhengzhou, Henan, China

**Keywords:** prostate cancer, radiofrequency ablation, anti-PD-1, immunity, combination therapy

## Abstract

**Background:**

This study seeks to investigate the potential synergistic effects of combining ultrasound-guided percutaneous radiofrequency ablation with anti-PD-1 therapy on prostate cancer, utilizing animal models.

**Methods:**

A mouse model of prostate cancer was established by subcutaneous injection of 1 × 10^6^ Myc-Cap cells on the right side of FVB mice. When the volume of the tumors reached about 400mm^3^, the mice were randomly divided into four groups and received corresponding intervention treatments. Among them, Group 1 was the blank control group, Group 2 was the simple anti-PD-1 treatment group, Group 3 was the simple radiofrequency ablation group, and Group 4 is the group that received percutaneous radiofrequency ablation combined with anti-PD-1 therapy under ultrasound guidance. The growth of the tumors was observed in mice after treatment in each group, tumor tissues were collected, and the immune status of the mice was analyzed through flow cytometry, immunohistochemistry, immunofluorescence, and other methods.

**Results:**

Compared with other treatment groups, ultrasound-guided percutaneous radiofrequency ablation combined with anti-PD-1 therapy significantly reduced the weight and volume of the tumors, demonstrating more effective tumor suppression. At the same time, combination therapy can promote the aggregation of T-cells within the tumor and increase the proportion of cytotoxic T-cells, increase the proportion of M1 macrophages and iNOS expression, and decrease the proportion of M2 macrophages and Arg expression in the local area of the tumors.

**Conclusion:**

Local ablation can improve the therapeutic effect of PD-1 monoclonal antibody. Our preliminary results suggest that ultrasound-guided percutaneous radiofrequency ablation, in combination with anti-PD-1 treatment, produces synergistic effects. These effects may be driven by changes in immune cell populations within the tumor’s immunosuppressive microenvironment.

## 1 Introduction

Prostate cancer (PCa) is the second most prevalent cancer in men and ranks as one of the leading causes of cancer mortality globally (1). According to clinical statistics for 2023, lung cancer, prostate cancer, and colorectal cancer are the leading causes of death in men, while in women, lung cancer, breast cancer, and colorectal cancer top the list of most common cancer-related fatalities. The incidence rate of PCa ranks the first in males, and its mortality rate is the second, just secondary to lung cancer. After two decades of decline in the incidence rate, the incidence rate of PCa has increased by about 3% every year from 2014 to 2019 (2), and in Asia, the incidence rate and mortality of PCa have both increased yearly (3). Although androgen deprivation therapy (ADT) has achieved outcomes as a traditional treatment for advanced prostate cancer, some tumors still eventually progress, so innovative treatment ideas and exploration of updated treatment methods are needed.

Tumor immunotherapy works by restoring the damaged immune system (4). Immune checkpoint blockade (ICB) is a type of immunotherapy that involves blocking the co-inhibitory pathways of the T-cells, namely T-cell checkpoints, to abolish their inhibition on T-cells, so as to alter the tumor’s immune suppressive microenvironment, and to trigger anti-tumor reactions (5). PD-1/PD-L1 inhibitors are among the most commonly used immunotherapeutic agents. They function by blocking the interaction between PD-1 and PD-L1, thereby restoring the cytotoxic activity of T lymphocytes (CTLs) and enhancing their ability to target and destroy cancer cells (6).These inhibitors have demonstrated significant therapeutic efficacy in treating solid tumors, including melanoma and non-small cell lung cancer (7, 8). However, as a “cold” tumor lacking immune cell infiltration within the tumor tissues (9), PCa has an immunosuppressive microenvironment and is less exposed to immunogenic antigens, thus showing poor responsiveness to ICB treatments (10–12).Moreover, the therapeutic efficacy of single-agent immune checkpoint inhibitors (ICIs) in clinical trials has been relatively modest (13). Therefore, transforming “cold” tumors into “hot” tumors, changing the microenvironment of the tumors, and increasing immune cell infiltration are the key to the treatment.The focus of prostate cancer immunotherapy has progressively shifted toward combination strategies, incorporating immunotherapy alongside standard treatments such as radiotherapy and chemotherapy, while also investigating the potential of effective dual immunotherapy approaches (11, 14–17).However, research exploring the combined efficacy of ultrasound-guided percutaneous radiofrequency ablation and anti-PD-1 therapy in prostate cancer is still limited.

Transcutaneous radiofrequency ablation (RFA) refers to the insertion of one or more radiofrequency electrodes into tumor tissues under the guidance of imaging equipment (ultrasound, CT, or MRI). Through high-frequency current, high-speed ion transport is induced within the cells, leading to localized high-temperature and tissue coagulation necrosis (18), accompanied by protein denaturation and tumor volume reduction.In addition to the thermal effect, there are studies reporting that RFA can activate the body’s immune system (19). Following tumor tissue ablation, the residual tissue *in situ*, along with a substantial influx of inflammatory immune cells and the release of inflammatory mediators, damage-associated molecular patterns (DAMPs), and other immune modulators, facilitates the infiltration and activation of immune cells within the tumor microenvironment. This cascade of events triggers immunogenic cell death, which subsequently promotes the activation of an antigen-specific immune response (20, 21).Therefore, this present study explores the potential synergistic effect of ultrasound-guided percutaneous radiofrequency ablation combined with anti-PD-1 therapy on prostate cancer through animal experiments, aiming to provide a new method for the treatment of prostate cancer.

## 2 Materials and methods

### 2.1 Establishment of the animal model

Myc-Cap is a cell line derived from a TRAMP transgenic prostate cancer mouse model, which expresses androgen receptors and is sensitive to androgens. The cells were kindly provided by Suzhou Haixing Biosciences Co., Ltd., and cultured in DMEM containing 10% fetal bovine serum and 1% penicillin/streptomycin.

6 to 8-week-old male FVB mice with normal immunity, which were used in this study, were purchased from the China SPF (Beijing) Biotechnology Co., Ltd. After the mice arrived, they were given one-week adaptation before conducting the experiment. During the experiment, all experimental mice were housed in the animal facility of the Experimental Animal Center at Zhengzhou University.

**Figure 1:**
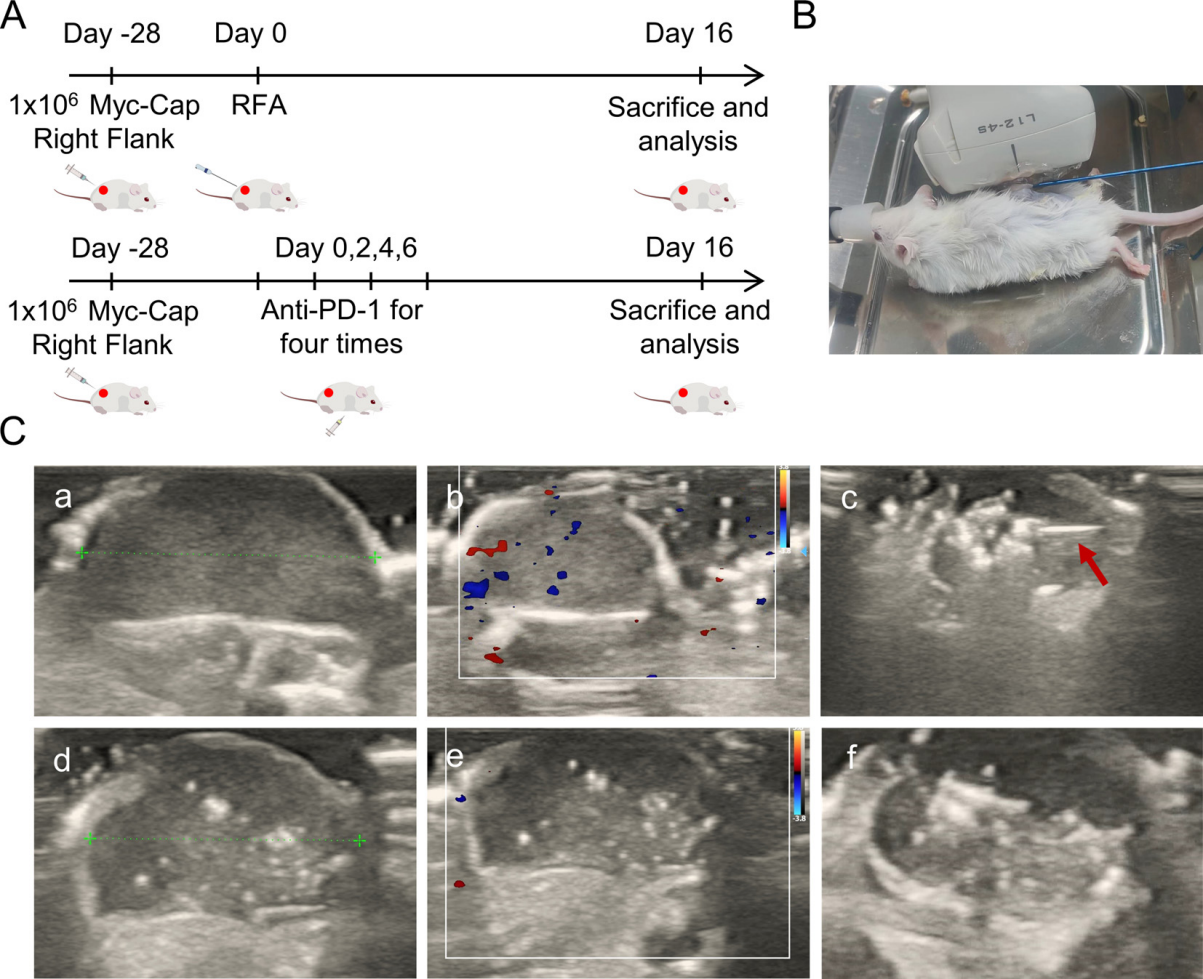
Study design protocol and ablation ultrasound findings. **(A)** Schematic diagram of the two treatment modalities. The FVB mice were injected with 1 × 10^6^ Myc-CaP cells on the right back. When the tumor volume reached around 400mm^3^, mice were randomized and corresponding interventions. PD-1 treatment starts at Day0 every other day for a total of four intraperitoneal injections. Ultrasound-guided percutaneous puncture ablation therapy was performed at Day0. Treatment effects were measured as tumor growth in mice. **(B)** The percutaneous radiofrequency ablation of the right back of mice. **(C)** Ultrasound findings and ablation procedure before and after ablation. The red arrow indicates the ablation needle. **(a)** Images of tumor threads measured before ablation in two-dimensional gray scale ultrasound mode. **(b)** Preablation tumor in color Doppler blood flow. **(c)** Ultrasound images during ablation, red arrows indicate the ablation needle during ablation. The strong echoic light mass is visible at the tip of the ablation needle. **(d)** Images of tumor threads measured after ablation in two-dimensional gray scale ultrasound mode. **(e)** After ablation the tumors were visualized in color Doppler flow. **(f)** Gross images of tumors in two-dimensional gray scale ultrasound mode after ablation.

Hair removal on the right side of the mice’s back and subcutaneous injection of 1 × 10^6^ cells were performed. Starting from the 7th day after cell injection, tumor size was measured every 2-3 days using a vernier caliper, and the volume was calculated according to the formula V=x · y^2^/2, where x refers to the longest diameter of the tumor, while y denotes the shortest diameter. When the volume of the tumor reached around 400mm^3^ (22), the mice were randomly grouped and corresponding intervention measures were carried out (**Figure 1A**). On the day when mice were separated into groups, ultrasound was used to record the volume of the tumors before and after treatment. Afterwards, the weight of the mice was recorded every other day, and the tumor size continued to be measured using a vernier caliper. Mice were euthanized by cervical dislocation when they were near death, when the tumor volume reached 2000 mm³, or when the longest tumor diameter exceeded 2 cm.The body weight of the mice was monitored throughout the experiment to provide an initial assessment of experimental safety.After euthanizing the mice, organ toxicity was evaluated by assessing liver and kidney function in each group, along with performing H&E staining on heart, liver, spleen, lung, and kidney tissues.

This research protocol was approved by the Welfare Ethics Committee of the Experimental Animal Center of Zhengzhou University (Zhengzhou, China, Protocol Number: ZZU-LAC2024031915), which was composed of animal welfare experts. The feeding and treatment of the mice were carried out in accordance with the approved animal protocol by the institution.

### 2.2 Experimental Procedures and Grouping

When the tumor volume of the mice reached approximately 400 mm³, they were randomly allocated into four groups for treatment. Among them, Group 1 was the blank control group, Group 2 was the simple anti-PD-1 treatment group, Group 3 was the simple radiofrequency ablation group, and Group 4 was the ultrasound-guided percutaneous radiofrequency ablation combined with anti-PD-1 treatment group.

Group 1: No intervention was performed.

Group 2: Mice were treated with InVivomAb Antimouse PD-1 (10 mg/kg, clone J43, Bioxcell, USA) for the first time starting from the day of grouping, and then the same treatment was administered every other day, a total of four intraperitoneal injections (**Figure 1A**).

Group 3: The LDRF-120S radiofrequency ablation (RFA) device (Mianyang, China) and the RFDJ01-111808007 radiofrequency electrode were used, with a working tip of 5mm and ablation parameters set to 10-15W. Mice were anesthetized with isoflurane inhalation, and their skin was pinched or their toes were stimulated without any response to ensure successful anesthesia. The fur in the local area of the mice was shaved, and they were then placed in a metal tray. The electrode pads were attached to the bottom of the tray and formed a circuit with the RF electrode. Hair removal and disinfection on the tumor site of the mice were performed, and ice packs were prepared to prevent excessive burns to the animals’ skin during surgery. Under ultrasound guidance, the radiofrequency needle was punctured into the tumor center for ablation (**Figure 1B**), and the ablation process was continuously monitored by ultrasound. According to the indications from the ultrasound, power was adjusted and the ablation frequency was increased. The ablation was ended when punctate hyperechoic areas occupied 50% of the tumor area. The ablation time was 60-80s, and the ablation temperature was 60-70°C (23, 24). After ablation, the area was observed for bleeding or other postoperative complications.

Group 4: On the day of grouping, ultrasound-guided percutaneous radiofrequency ablation and the first intraperitoneal injection of Anti-mouse PD-1 were performed under the same operating and experimental conditions as the “simple anti-PD-1” group and the “simple radiofrequency ablation” group. The remaining three doses were administered every other day.

### 2.3 Anatomical analysis of mice tumor tissues, blood, and major organs

#### 2.3.1 Flow cytometry

After the mice were euthanized, the tumor and spleen were removed. After digesting the tumor tissues, they were ground and washed together with the spleen to prepare a single-cell suspension. After staining with the corresponding antibody, a 100 μl suspension was prepared and stored in the dark for analysis by flow cytometry.

In order to stain regulatory T-cells, anti-CD25-APC and anti-CD4 FITC were first used to stain the antibodies at the surface of the cells. After further cell fixation, membrane rupture, and nucleus rupture, anti-Foxp3-PE staining was performed. The staining of M2 macrophages was carried by first staining the antibodies at the surface of the cells, and after fixing and permeabilizing the cells, they were stained with anti-CD206-APC. The antibodies used for flow cytometry analysis, namely anti-CD3-PE (catalogue no.100308), anti-CD4-FITC (catalogue no.130308), anti-CD8-APC (catalogue no.100712), anti-foxp3-PE (catalogue no.126403), anti-CD25-APC (catalogue no.102011), Cell Activation Cocktail (catalogue no.423303), anti-Granzyme B Recombinant-PE (catalogue no.372207), anti-F4/80-FITC (catalogue no.123107), anti-CD11b PE (catalogue no.101207), anti-CD45-PerCP (catalogue no.103131), anti-CD206-APC (catalogue no.141707), and anti-CD86-APC (catalogue no.105011), were all purchased from the BioLegend Corporation (USA), and all data were analyzed using the FlowJo software.

#### 2.3.2 Immunohistochemical staining

The tumor tissues were fixed in paraformaldehyde, then embedded in paraffin. Serial sections were prepared, deparaffinized with xylene, and dehydrated using ethanol. Antigen repair was performed, and endogenous peroxidase was blocked. Then primary antibodies (F4/80, CD4^+^, CD8^+^, PD-1, CD31, KI67) were added and incubated overnight at 4 °C. After secondary antibodies were added and incubated at room temperature, DAB staining was performed. Under the microscope, the staining result was observed. Then they were counterstained with hematoxylin, and treated by the hematoxylin differentiation solution and the hematoxylin bluing solution to “blue”. After dehydration and sealing, the films were read under a microscope.

#### 2.3.3 Immunofluorescence technique

After dewaxing the paraffin sections of tumor tissues to water, antigen repair, circling, and serum blocking, the primary antibody was added, and then incubated overnight at 4°C. Afterwards, incubation at room temperature was carried out for 50 minutes, and corresponding tsa dye was added. After microwave treatment, the secondary antibody was added, and then incubated overnight at 4°C, followed by the addition of the fluorescently labeled secondary antibody, washing, DAPI counterstaining, quenching of spontaneous fluorescence, sealing, and image acquisition.

#### 2.3.4 qPCR

After tumor tissues were removed, they were stored in liquid nitrogen. Total mRNA was extracted using the Trizol method, followed by cDNA synthesis with a q-PCR kit (Wuhan Sevier). Quantitative real-time PCR was performed using SYBR Green PCR Master Mix and specific primers to measure the mRNA levels of Arg and iNOS. Gene expression was analyzed using the 2^-△△C^ method.

#### 2.3.5 ELISA

Mouse blood was collected using the orbital blood collection method. The whole blood sample was left at room temperature for 15 minutes and centrifuged (3,000 rpm/min, 15 min), and the supernatant was collected. Following the instructions of the reagent kit (Wuhan Sevier), the concentrations of IL-12, TNF-α, IL-10, TGF-β, IL4, and TNF-γ were detected.

### 2.4 Statistical analysis

Statistical analysis was conducted using the Graph Pad Prism 8.0 software. The significant difference in unpaired tails was analyzed using Student’s t-test. All experimental results were repeated at least 3 times and are presented as as mean ± standard deviation (SD). A p-value of <0.05 was considered statistically significant. In the figures, p-values are denoted as * P<0.05, ** P<0.01, *** P<0.001, and **** P<0.0001.

## 3 Results

### 3.1 Ultrasound manifestations of tumors before and after percutaneous radiofrequency ablation

On the day of random grouping of the mice, ultrasound evaluation was performed. When no intervention measures were taken, the tumors of each group of mice showed elliptical hypoechogenicity under two-dimensional grayscale ultrasound (**Figure 1C, a**), with uniform echoes, clear boundaries, and regular morphology. Color Doppler flow imaging (CDFI) (**Figure 1C, b**) showed dotted blood flow signals around and in the center of the tumors. After the first intraperitoneal injection of the drug in the mice of the simple anti-PD-1 treatment group, there was no significant difference in tumor ultrasound findings. Under ultrasound guidance, radiofrequency ablation was performed on the tumors of mice from the simple radiofrequency ablation group and he ultrasound-guided percutaneous radiofrequency ablation combined with anti-PD-1 treatment group. During ablation, dynamic spot/plaque-like hyperechoic clusters appeared at the tip of the ablation needle within the tumors (**Figure 1C, c**). As the ablation needle was moved, the area of hyperechoic signals gradually expanded. When the hyperechoic area occupied more than 50% of the tumor area, ablation was terminated. After ablation, the hyperechoic gas gradually dissipated, but focal or patchy hyperechoic areas remained within the tumor on the two-dimensional grayscale ultrasound (**Figure 1C, d, f**). Color Doppler flow imaging (CDFI) revealed no significant blood flow signals in the tumor’s peripheral or central regions (**Figure 1C, e**), showing a marked contrast with the pre-ablation ultrasound patterns.Gross observation revealed slight yellowing of the skin around the tumors.

### 3.2 Tendency of tumor volume changes in each group after treatment

**Figure 2:**
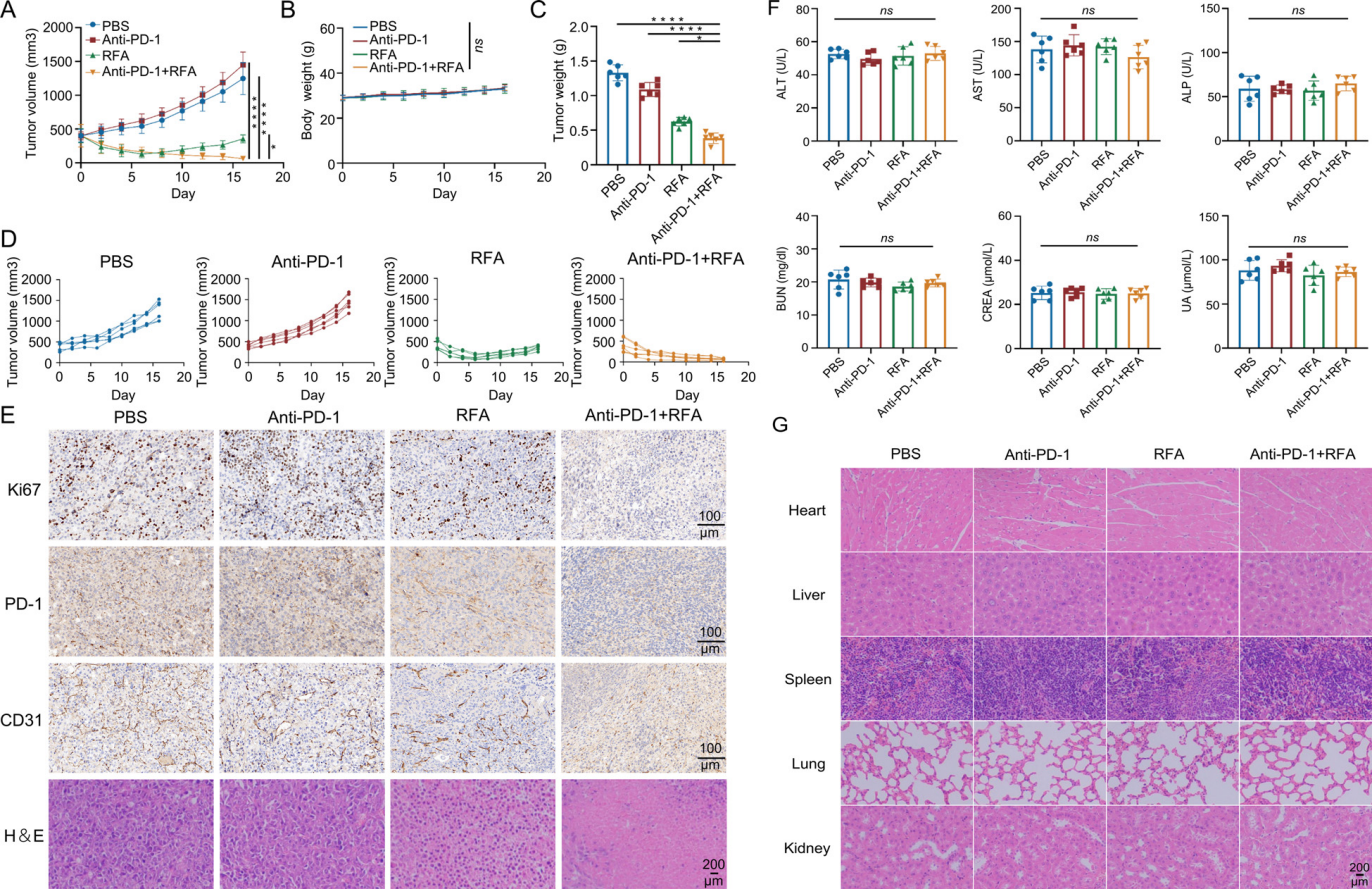
Tendency of tumor volume changes in each group after treatment and biological safety analysis. **(A)** Curves of the mouse mean tumor volume over time (n=6). **(B)** Body weight changes of the mice during the experiment (n=6). **(C)** Tumor weights were compared at the experimental endpoint (n=6). **(D)** Curcurve of individual tumor volume over time within each group (n=6). **(E)** Mouse tumors were subjected to immunohistochemistry for Ki67, CD-1, and CD31 and analysis of tissue sections. **(F)** Biochemical assessment of liver and kidney function in mice (n=6). **(G)** Pathological sections of vital internal organs in mice. Data were shown as the mean ± SD. ns, no significance, *P < 0.05 and ****P < 0.0001.

The growth rate and tendency observed in the simple anti-PD-1 treatment group and the blank control group were similar, and the volume of the tumors continuously increased (**Figure 2A**). After treatment, the volume of the tumors decreased in the simple radiofrequency ablation group and the ultrasound-guided percutaneous radiofrequency ablation combined with anti-PD-1 treatment group. On the eighth day after treatment, the volume of the tumors in the simple radiofrequency ablation group gradually increased, while tumor growth was slow and stable without significant changes in the ultrasound-guided percutaneous radiofrequency ablation combined with anti-PD-1 treatment group (**Figure 2A, C, D**), indicating the good anti-tumor effect of the combined therapy. H&E staining and Ki67 staining of the tumor tissues showed that in mice subjected to combination therapy, a reduction in tumor cell count was observed, along with a downregulation of Ki67 expression. (**Figure 2E**). In addition, PD-1 and CD31 staining showed that in tumor tissues of mice treated with combination therapy, a marked downregulation of PD-1 and CD31 expression was observed (**Figure 2E**). Meanwhile, there were no significant abnormalities or differences in the mice’s body weight, organ toxicity, and serum biochemistry among the groups (**Figure 2B, F, G**).

### 3.3 Characteristics of immune T cell infiltration changes (such as CD8^+^ and CD4^+ ^T cells) in each group after treatment

**Figure 3:**
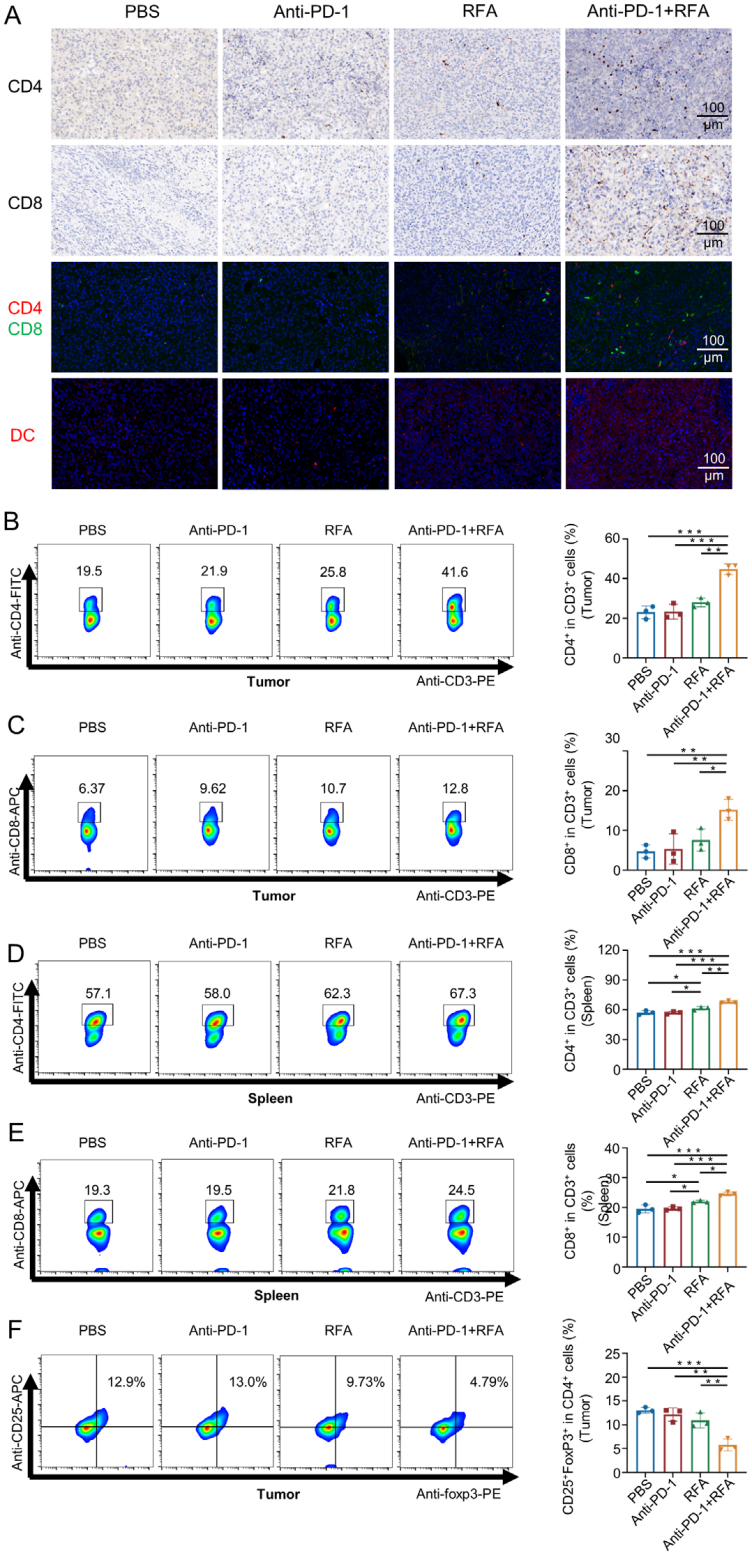
Characteristics of immune T cell infiltration changes (such as CD8^+^ and CD4^+^ T cells) in each group after treatment. **(A)** Immunohistochemical and immunofluorescence analysis of CD4^+^ and CD8^+^ T cells, and immunofluorescence analysis of dendritic cells in mouse tumors. **(B-F)** Flow analysis of mouse tumors and spleen-associated T cells (n=3). Data were shown as the mean ± SD. *P < 0.05, **P < 0.01, and ***P < 0.001.

The types of T-cells mediating tumor suppression in prostate cancer models were detected through immunohistochemistry, immunofluorescence staining, and flow cytometry. the blank control group, the simple anti-PD-1 treatment group, and the simple radiofrequency ablation group all showed limited CD4 and CD8 fluorescence signals and weak immunohistochemical staining, while the ultrasound-guided percutaneous radiofrequency ablation combined with anti-PD-1 treatment group showed strong CD4^+^ and CD8^+^T fluorescence signals and immunohistochemical staining within the tumors (**Figure 3A**). Flow cytometry analysis demonstrated an increased proportion of CD4^+^ and CD8^+^ T cells in the tumor tissues of mice treated with the combination therapy (**Figure 3B, C**). Furthermore, the frequency of CD4^+^ and CD8^+^ T cells in the spleen showed a corresponding trend. (**Figure 3D, E**). Also, the proportion of the local immunosuppressive regulatory T-cells (Treg) In the combination therapy group was lower compared with the other three groups (**Figure 3F**).Meanwhile, the fluorescence signal of local dendritic cells (DCs) was observed (**Figure 3A**).

### 3.4 Characteristics of changes in M1, M2, and related factors in each group after treatment

**Figure 4:**
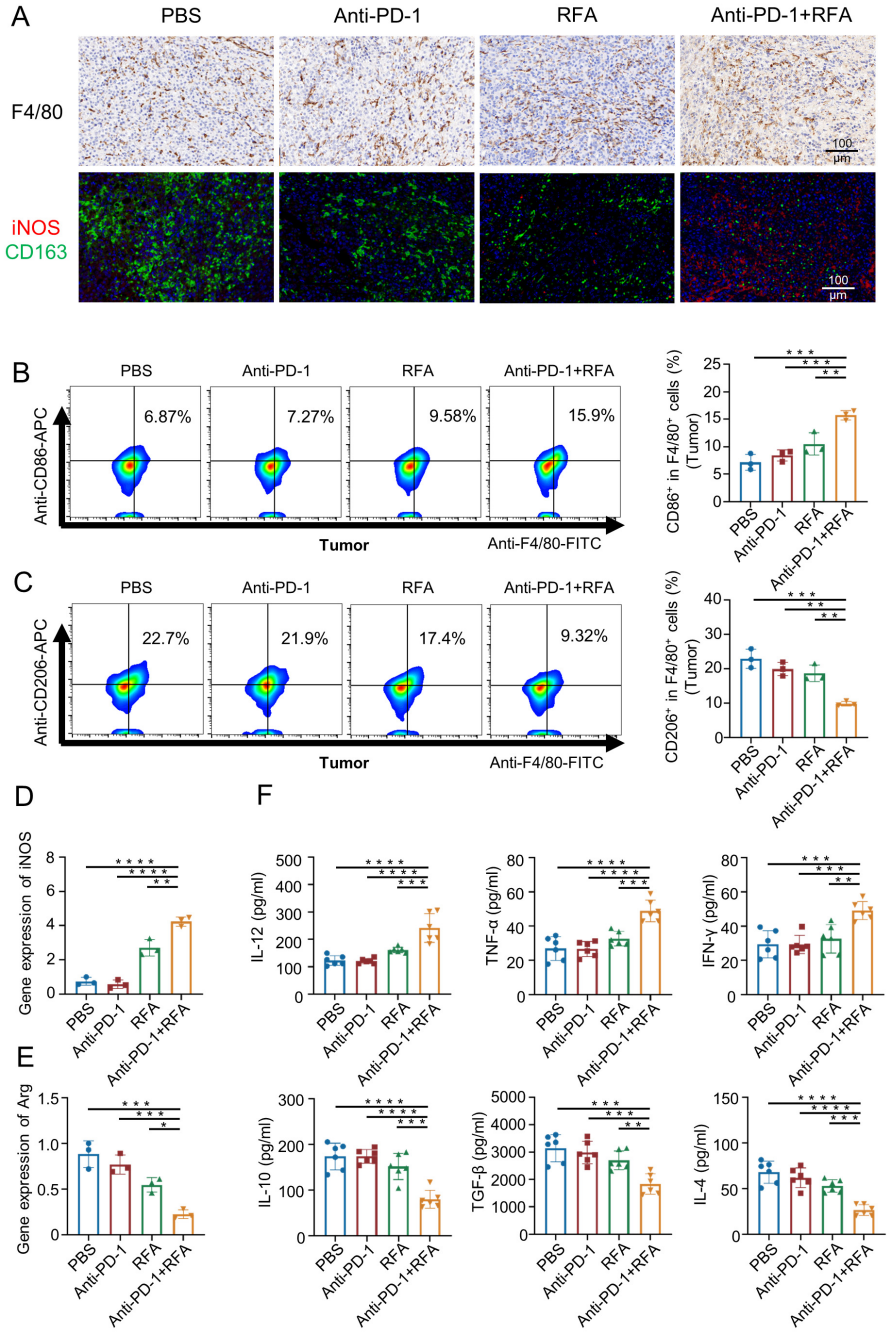
Characteristics of changes in M1, M2, and related factors in each group after treatment. **(A)** Immunohistochemistry and immunofluorescence analysis of macrophage cells from mouse tumors. **(B, C)** Flow analysis was performed on mouse tumor macrophages. (n=3). **(D)** iNOS expression in local tumor macrophages was analyzed (n=3). **(E)** Arg expression in local tumor macrophages was analyzed (n=3). **(F)** For analyzing the levels of cytokines in the blood (n=6). Data were shown as the mean ± SD. *P < 0.05, **P < 0.01, ***P < 0.001, and ****P < 0.0001.

The qPCR results showed that compared with the other three groups, the mRNA level of inducible nitric oxide synthase (iNOS) associated with M1 macrophages was significantly increased in the ultrasound-guided percutaneous radiofrequency ablation combined with anti-PD-1 treatment group (**Figure 4D**), while the mRNA level of arginine synthase (Arg) associated with M2 macrophages was significantly decreased (**Figure 4E**). Thus, macrophage infiltration at the tissue level was further assessed using immunohistochemistry, immunofluorescence staining, and flow cytometry.The F4/80 immunohistochemical staining (**Figure 4A**) results showed extensive infiltration of macrophages in tumors of all groups. However, immunofluorescence staining for iNOS and CD163 (**Figure 4A**), along with flow cytometry results (**Figure 4B, C**), revealed that tumors in the blank control group, anti-PD-1 treatment group, percutaneous radiofrequency ablation group, and combination therapy group exhibited an increased proportion of M1 macrophages and a decreased proportion of M2 macrophages.The peripheral blood test results of the mice (**Figure 4F**) showed that compared with the other three groups, the pro-inflammatory cytokines Interleukin-12 (IL-12), Tumor Necrosis Factor-alpha (TNF-α), and Th1 cytokine Interferon-γ (IFN-γ) secreted by the M1 macrophages in the mice treated with the combination therapy increased, while the cytokines Interleukin-10 (IL-10), transforming growth factor beta (TGF-β), and Th2 cytokine Interleukin-4 (IL-4) secreted by the M2 macrophages decreased.

## 4 Discussion

Immune checkpoint inhibitors are currently a hot topic in tumor immunotherapy research, but their application in the PCa,a “cold” tumor (25), shows limitations (26). Among them, anti-PD-1 therapy in combination with multiple treatment methods has shown good therapeutic effects on PCa (27–29), but there is relatively little research on its combination with radiofrequency ablation as a treatment modality. According to the research, radiofrequency ablation can help disrupt the immune suppressive microenvironment of the tumor, promote antigen presentation, and improve the efficacy of immunotherapy (21).Therefore, a mouse PCa model was used to evaluate the potential synergistic effect of ultrasound-guided percutaneous radiofrequency ablation and anti-PD-1 combination therapy in this study, and it was demonstrated that this potential synergistic effect may depend on the infiltration of more T-cells and the transformation of M2 macrophages into M1 macrophages.

Consistent with previous studies, in this study, anti-PD-1 therapy alone did not show a significant inhibitory effect on PCa, with poor therapeutic efficacy (30, 31), and did not significantly alter the tumor’s suppressive microenvironment, which also suggests the necessity of combination therapy.

During ultrasound-guided radiofrequency ablation, it was found that the ultrasound images of the tumors changed before and after ablation, and the blood flow signal of the tumors weakened. Compared with the other two groups, the tumor volume of mice in the radiofrequency ablation only group and the combination therapy group showed a decreasing trend in the early stage of treatment. The process of cell heating induces almost immediate and irreparable cell damage, leading to the coagulative necrosis of the tissues (18). Therefore, it is speculated that the thermal effect generated during ablation can reduce tumor volume and lower tumor burden. Although only radiofrequency ablation was used as the local ablation method in this study, we speculate that other thermal ablation treatments, such as microwave ablation, high-intensity focused ultrasound ablation, laser ablation, etc., may also effectively reduce tumor burden and show similar therapeutic effects.

The results of this study showed significant differences in tumor volume changes between mice treated by radiofrequency ablation alone and those treated by combination therapy. The simple radiofrequency ablation treatment group showed a decreasing trend in tumor volume for about eight days only, followed by an increasing trend, while the combination therapy group showed a more gradual and stable change in tumor volume. Meanwhile, compared with the combination therapy group, the tumor slices of mice treated with radiofrequency ablation only showed higher expression of Ki67, heavier staining, and cell nuclei division, indicating active cell division. Moreover, local infiltration of immune cells at the tumor was reduced. This is consistent with previous research results, which showed that residual tumor cells grow faster after radiofrequency ablation (32, 33). The area of thermal damage is divided into three regions, namely the central high-temperature zone, the sub-lethal temperature transition zone, and the surrounding normal tissues. In the transition zone, tumors suffer reversible damage and ultimately survive, leading to rapid proliferation, invasion, and metastasis of the residual tumor cells under an activated state (34). In addition, the thermal stimulation and low oxygen environment generated by radiofrequency ablation can also stimulate endothelial cells and promote the growth of residual tumors (35). In this study, the expression of CD31 in the tumors of mice treated with radiofrequency ablation alone showed no significant difference compared with the blank control group, indicating that the tumors have abundant blood vessels, which is also consistent with previous research results. Due to various factors such as tumor size and location, complete ablation cannot be guaranteed each time, and the remaining tumor tissues show the potential of recurrence or even faster growth. Therefore, it is necessary to improve treatment efficacy through combination therapy.

In mice treated with ultrasound-guided percutaneous radiofrequency ablation and anti-PD-1 combination therapy, there was no significant increase in tumor volume and no recurrence, indicating that the combination of the two methods shows better anti-tumor effects. A clinical study on recurrent hepatocellular carcinoma also indicated similar results, which concluded that the combination therapy of anti-PD-1 and RFA was superior to RFA alone (36). In this study, compared with the other three groups, mice that received combination therapy showed a decrease in PD-1 expression in the tumor area, an increase in the proportion of CD4^+^T cells,CD8^+^T cells and DCs, and a decrease in the proportion of Treg cells, indicating an improvement in the immunosuppressive microenvironment.The release of tumor antigens following ablation facilitated dendritic cell maturation, thereby triggering the initiation and activation of antigen-specific effector T cells, representing the second phase of the anti-tumor immune response (10).Some clinical studies have also indicated similar results (37).However, this study did not assess T cell activity, and future research should focus on further investigating alterations in T cell function, such as the evaluation of cytotoxic T lymphocyte (CTL) activity using B^+^ CTL assays.

Tumor associated macrophages (TAMs) are an important component of the tumor microenvironment (TME), exhibiting classic anti-tumor activation of the M1 type or alternative tumorigenic activation of the M2 type (38). The proportion of M2 TAMs in TME is relatively high, which can promote tumor growth, invasion, and metastasis by secreting various active substances (39, 40). M1 macrophages, through iNOS, produce NO that inhibits cancer cell growth, while M2 macrophages produce ornithine that promotes cancer cell growth through Arg (41). In this study, compared with the other three groups, the number of M1 macrophages and iNOS expression in the tumor area of mice treated with combination therapy increased, indicating that M1 macrophages exhibited a certain anti-tumor role, while M2 macrophages showed the opposite trend, with a decrease in number and Arg expression, suggesting the transformation of M2 into M1. At the same time, CD31 staining showed a decrease in CD31 expression, indicating a reduction in tumor vascular distribution, thus a decrease in the ability of metastasis and proliferation. At the cytokine level, it was observed in this study that cytokines IL-12 and TNF–α released by M1 TAMs increased, promoting T-cell proliferation and survival. At the same time, the secretion of IFN-γ by Th1 cells increased in serum, enhancing immune function. However, IL-10 and IL-4 decreased, suggesting that polarization of M2 TAMs may have been inhibited (42). In our study, the level of TGF-β (41) secreted by M2 TAMs in serum was low, indicating that tumor development and metastasis were inhibited to some extent, increasing the activation of M1 macrophages. As an immunosuppressive cytokine, IL-10 can block the co-stimulatory pathway required for activating CTLs and promote the differentiation of Treg cells; TGF-β can induce Treg cells from their precursors (37). The decrease in the proportion of Treg cells in this study may be related to the decrease in IL-10 and TGF-β levels.

Therefore, the combination of ultrasound-guided percutaneous radiofrequency ablation and anti-PD-1 therapy synergistically inhibited tumor progression, likely by altering immune cell populations within the immunosuppressive tumor microenvironment.Radiofrequency ablation technology is already mature in the treatment of liver cancer, but its application in the treatment of PCa is relatively limited. The present study used radiofrequency ablation combined with immunotherapy for the treatment of PCa, which is a relatively new attempt. At the same time, this study has not only explored the characteristics of T-cell changes, but also conducted preliminary analysis and exploration on TAMs, resulting in good results.Ablation therapies have shown the ability to induce tumor antigen-specific immune responses in preclinical and clinical models, potentially transforming prostate cancer into an immunologically “hot” tumor (10).The combination of this approach with PD-1 inhibitors in clinical practice holds considerable promise.However, systemic administration of medication may be associated with inevitable adverse reactions. As an alternative, intratumoral administration of immunotherapy (ITIO), which involves directly injecting immunotherapeutic agents into target lesions, can significantly reduce the risk of systemic side effects (43).

This study also has certain limitations. Firstly, due to the longer period it takes to build castration-resistant prostate cancer mice, this group is not set up. Currently, castration-resistant prostate cancer (CRPC) shows a high mortality and metastasis recurrence rate, which is clinically challenging.It is critical to evaluate the effectiveness of its combination therapy.Moreover, this study lacks long-term follow-up data and does not assess the durability of the treatment effects or the “memory” of the immune cells in the mice.Therefore, in future studies, we plan to expand the scope by incorporating additional animal models, including CRPC and rechallenge models (tumor rechallenge 25-30 days after treatment). We will also monitor long-term data to enhance the clinical relevance, scientific robustness, and rigor of the findings.Secondly, this experiment only provides an initial exploration of the potential causes underlying the combination treatment effect. In future studies, we will further investigate the causal relationship by exhausting specific immune cells and focus on the Akt/mTOR pathway to better understand the anti-tumor mechanisms involved. Last, other immune checkpoint inhibitors may be more effective than anti-PD-1 therapy, but we only combined with anti-PD-1 therapy and did not combine with other drugs. Similarly, we only used radiofrequency ablation as the ablation method, and further research is needed to determine whether other thermal ablation methods such as microwave ablation and laser ablation show better therapeutic effects. Therefore, in the future, we will try more combinations of immunotherapeutic drugs and thermal ablation methods to explore the best treatment strategy.

In summary, this study concludes that the application of local radiofrequency ablation can not only reduce tumor burden, but also stimulate the body to produce inflammatory reactions, stimulate anti-tumor immunity, and transform the tumor’s environment from an immunosuppressive state to an immuno-activated state, thereby improving the therapeutic effect of the PD-1 monoclonal antibody. It is preliminarily confirmed that ultrasound-guided percutaneous radiofrequency ablation combined with anti-PD-1 therapy for PCa shows a synergistic effect, which may be related to the alteration of immune cells within the inhibitory immune microenvironment.

## Data Availability

The original contributions presented in the study are included in the article/supplementary material. Further inquiries can be directed to the corresponding author.
